# Double emulsions as delivery systems for iron: Stability kinetics and improved bioaccessibility in infants and adults

**DOI:** 10.1016/j.crfs.2022.04.003

**Published:** 2022-04-14

**Authors:** Bruno Sérgio Toledo Barbosa, Edwin Elard Garcia-Rojas

**Affiliations:** a Programa de Pós-graduação em Ciência e Tecnologia de Alimentos (PPGCTA), Universidade Federal Rural de Rio de Janeiro (UFRRJ), Rodovia BR 465, Km 7, Seropédica, RJ, 23890-000, Brazil; b Laboratório de Engenharia e Tecnologia Agroindustrial (LETA), Universidade Federal Fluminense (UFF), Av. Dos Trabalhadores, 420, 27255-125, Volta Redonda, RJ, Brazil

**Keywords:** Tara gum, Whey protein, Microencapsulation, Minerals, Bioaccessibility

## Abstract

Iron deficiency is one of the main causes of anemia in the world, especially in children and women, so food fortification through microencapsulation is a viable alternative to combat this deficiency. The present work aimed to encapsulate iron in a water-in-oil-in-water double emulsion (W_1_/O/W_2_), which was formed with whey protein isolate and polyglycerol polyricinoleate as the emulsifying agents, tara gum as a thickening agent, and sucrose as an osmotic active substance. The double emulsion formed with 12% whey protein isolate, 0.8% tara gum, and 2% sucrose presented high encapsulation efficiency (96.95 ± 1.00%) and good stability (up to 7 days). Additionally, after the in vitro gastrointestinal simulations, the bioaccessibility was high for adults (49.54 ± 5.50%) and infants (39.71 ± 2.33%). Finally, the study show that double emulsions can form stable systems with high iron bioaccessibility even in infant gastric systems, which indicates the possibility of using double emulsions to fortify food with iron.

## Highlights

• Stable double emulsions were obtained using WPI and PGPR as emulsifiers.
• Tara gum ensured an increase in the general stability of the emulsion.
• High bioaccessibility of iron were obtained for adults and infants.
• Emulsions are presented as a potential alternative to be used in iron-fortified food.

## Graphical abstract

**Figure:**
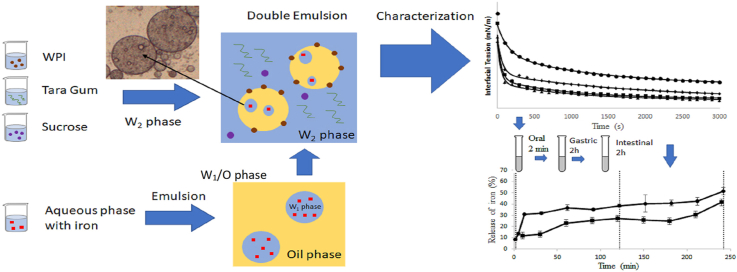

## 1 Introduction

Anemia is a clinical condition that presents a low serum hemoglobin concentration; among the main causes, iron deficiency can be highlighted. This condition affects many people, especially women and children. For example, approximately 40% of children in the world suffer from anemia (Shubham et al., 2020; Sun et al., 2021). To solve this health problem, food fortification using iron can be an alternative. However, direct food fortification with iron can generate unwanted results, such as changes in color, odor, and taste. In addition, iron has major inhibitors such as polyphenols, which can almost nullify its absorption in the human body, and calcium and calcium salts may also negatively affect the iron absorption (Hurrell et al., 1999; Lönnerdal, 2010; Gera et al., 2012; Cian et al., 2021). To minimize these inconveniences, it may be necessary to use technologies such as microencapsulation, where a substance is coated with an outer layer that protects it from various external factors such as light and temperature. The outer coating may also mask some organoleptic properties such as taste, odor, and sometimes color (Ye et al., 2018).

Several authors microencapsulated iron using different methods, including spray-drying (Bryszewska, 2019), hydrogels (Kazemi-Taskooh and Varidi, 2021), and simple (Prichapan et al., 2018) or double emulsions (Choi et al., 2009; Duque-Estrada et al., 2019; Ilyasoglu Buyukkestelli and El, 2019; Naktinienė et al., 2021). Emulsions can be defined as a homogeneous mixture of two immiscible liquids, where the dispersed phase is spread as a droplet in the continuous phase. Emulsions can be simple, such as water dispersed in oil or oil dispersed in water. They can also be multiples, e.g., double emulsion water-in-oil-in-water (W_1_/O/W_2_), where the first phase is a water-in-oil emulsion dispersed in the aqueous phase (Lu et al., 2016). In several studies, double emulsions are used as delivery systems in many liquid foods such as beverages (Aditya et al., 2015), milk and dairy products (Chang et al., 2016; Hernández-Marín et al., 2016).

Double emulsions are suitable for encapsulating water-soluble ingredients such as iron but are less stable than single emulsions (Muschiolik and Dickinson, 2017). Since they are thermodynamically unstable systems, emulsions tend to separate their phases by physicochemical mechanisms such as gravity separations such as creaming and sedimentation, and aggregation processes such as flocculation and coalescence. Therefore, it is necessary to use stabilizers that can prevent phase separation (Fredrick et al., 2010; Hu et al., 2017).

Among various components of emulsions, emulsifying agents are substances that adsorb at the oil-water interface and prevent drop aggregation. These agents can be hydrophobic or hydrophilic, depending on with which part they have more affinity (McClements and Jafari, 2018; Mettu et al., 2018). Proteins are highly effective emulsifying agents because they can adsorb on the surface. Additionally, they are electrically charged (away from the isoelectric point), which generates electrostatic stability in the emulsion drops (Dickinson, 2010). Whey proteins are a by-product of cheese production and commonly used as emulsifiers, foaming, and gelling agents. Whey protein isolate (WPI) consists mainly of α-lactalbumin, whey albumin, and β-lactoglobulin. These proteins have good nutritional qualities, are inexpensive, and have emulsifying properties similar to sodium caseinate (Dickinson, 2011; Bai et al., 2019). As a hydrophobic emulsifier, polyglycerol polyricinoleate (PGPR) is formed by esterifying glycerol polymerization with condensed castor oil and used to form water-in-oil emulsions (Mettu et al., 2018; Okuro et al., 2019).

In addition to emulsifiers, polysaccharides can be used as thickening agents because they increase the viscosity of the continuous phase of an emulsion, hinder the interaction among the drops, and prevent the droplets from aggregating (Dickinson, 2010). Tara gum is a galactomannan extracted from *Caesalpinina spinos*, native to Peru. This polysaccharide presents a structure formed by mannose and galactose in a 3:1 ratio. Tara gum is a neutral polysaccharide; therefore, it does not have a positive or negative electrostatic charge, and there is no attraction or repulsion with other electrically charged biopolymers (Borzelleca et al., 1993; Santos and Garcia-Rojas, 2021). Tara gum can bind to water and increase the system viscosity without forming a gel; therefore, its use as a thickening agent can be considered satisfactory (Wu et al., 2009, 2018). However, few studies have studied galactomannan as a thickening agent in emulsions (Kaltsa et al., 2016).

Although studies show that iron can be encapsulated with good efficiency and stability, the effect of tara gum as a thickener in a double emulsion must be examined. In addition, studies on iron bioaccessibility are scarce, and no studies were found to identify the iron bioaccessibility in double emulsions in simulated infant gastrointestinal systems. Thus, this study aimed to form and characterize double emulsions for iron encapsulation and study their bioaccessibility for adults and infants through in vitro gastrointestinal simulation.

## 2 Materials and methods

### 2.1 Material

Tara gum was obtained from El Sol SAC (Lima, Peru), PGPR was obtained from the local market (Sao Paulo, Brazil), Soybeam oil was obtained from the local market (Rio de Janeiro, Brazil), and whey protein isolate (WPI) was obtained from Glanbia Nutritionals (Fitchburg, USA). Sucrose, iron sulfate heptahydrate (215422), hydroxylamine hydrochloride, 3-(2-pyridyl)-5,6-diphenyl-1,2,4-triazine-p, p-disulfonic acid monosodium salt hydrate (160601), hydrochloric acid, sodium hydroxide, α-amylase (A33403), porcine pepsin (P6887), porcine pancreatin (P7545), porcine bile extract (B8631) and sodium azide were obtained from Sigma–Aldrich® (St. Louis, USA). Potassium chloride (1526) was obtained from Dinâmica Química (São Paulo, Brazil), Magnesium chloride (2014.01. AG) and ammonium carbonate (C1003.01. AF) were obtained from Synth (São Paulo, Brazil). Sodium chloride (310), sodium bicarbonate (302), and potassium phosphate (365) were obtained from Isofar (Rio de Janeiro, Brazil). Calcium chloride (CC06568RA) was obtained from Êxodo Cietífica (São Paulo, Brazil). All reagents were of analytical grade. The water used was ultrapure with a conductivity of 0.05 μS/cm (Master System P&D, Gehaka, Brazil).

### 2.2 Formation of double emulsion (W_1_/O/W_2_)

The double emulsion was formed in a two-step process adapted by Duque-Estrada et al. (2019) and Ilyasoglu Buyukkestelli and El (2019).

#### 2.2.1 Formation of a simple emulsion (W_1_/O)

The W_1_/O emulsion was formed with an aqueous phase (W_1_) with 0.8% w/w of iron sulfate, and soybean oil (O) contained 5.0% w/w PGPR. Each phase was obtained by mixing the constituents at 200 rpm for 30 min at room temperature, followed by 30 min of rest. The emulsion was prepared by mixing the oil phase and aqueous phase at a ratio of 4:1 using an UltraTurrax T25 homogenizer (IKA, Germany) at 15,000 rpm for 4 min.

#### 2.2.2 Formation of double emulsions (W_1_/O/W_2_)

The external aqueous phase (W_2_) was prepared with WPI and tara gum mixed solutions at different concentrations: 8, 12, or 16% w/w and 0, 0.4, or 0.8% w/w for WPI and tara gum. These solutions were prepared in the presence or absence of sucrose (0 and 2% w/w) as an osmotic active substance. Different samples evaluated from the external aqueous phase are shown in Table 1. To produce W_1_/O/W_2_ emulsions, the W_2_ and W_1_/O phases were mixed (4:1 ratio) and homogenized with UltraTurrax at 14,000 rpm for 3 min at room temperature. Sodium azide (0.01% w/w) was also added to W_2_ as a bacterial inhibitor.

**Table 1:** **- C**omponents of the external aqueous phase (W_2_) of the double emulsions (W_1_/O/W_2_).

Sample∗	WPI (%)	Tara Gum (%)	Sucrose (%)
A1	8.0	0.0	0.0
A2	12.0	0.0	0.0
A3	16.0	0.0	0.0
A4	8.0	0.4	0.0
A5	12.0	0.4	0.0
A6	16.0	0.4	0.0
A7	8.0	0.8	0.0
A8	12.0	0.8	0.0
A9	16.0	0.8	0.0
A10	8.0	0.0	2.0
A11	12.0	0.0	2.0
A12	16.0	0.0	2.0
A13	8.0	0.4	2.0
A14	12.0	0.4	2.0
A15	16.0	0.4	2.0
A16	8.0	0.8	2.0
A17	12.0	0.8	2.0
A18	16.0	0.8	2.0

∗ The samples contain the same composition in the internal phase W_1_/O.

#### 2.2.3 Osmotic active substance

Sucrose was used as the osmotic active substance (OAS). Its concentration was used to equalize the osmotic pressures between the internal and external phases of water in the double emulsion, which prevented the transport of water and subsequent instability in the system. Thus, the ideal gas equation is adopted to identify the equilibrium osmotic pressure (Bonnet et al., 2009; Simiqueli et al., 2019) by Equation (1):

(1) $$C_{suc}=\;C_{Fe}+C_{SO_{4}}$$

where *C*_*suc*_ is the sucrose concentration, and *C*_*Fe*_ and *C*_*SO4*_ are the molar concentrations of iron sulfate.

### 2.3 Kinetic stability of double emulsions (W_1_/O/W_2_)

The kinetic stability of the emulsions was determined according to the methodology described by Ilyasoglu Buyukkestelli and El (2019). Ten milliliters of double emulsions were placed in transparent tubes and kept in a Shaker SL222 incubator (Solab, Brazil) at 25 °C for 7 days. The height of the transparent layer was observed at the bottom of the tube during the 7 days, and the creaming index of the emulsion was obtained by Equation (2):

(2) $$CI\left(\%\right)=\left(H_{c}/H_{t}\right)\times 100$$

where CI (%) is the creaming index, H_*c*_ is the height of the clear layer, and H_*t*_ is the total height of the system.

### 2.4 Encapsulation efficiency of iron in double emulsions (W_1_/O/W_2_)

The encapsulation efficiency (EE) was measured in the samples with the best kinetic stability during 7 days of storage. EE was determined from the amount of iron in the outer aqueous phase (W_2_), which was calculated by the adapted ferrozine chelation method (Choi et al., 2009; Prichapan et al., 2021). Ten milliliters of double emulsion was centrifuged with an 80-2B centrifuge (Centribio, Brazil) at 4000 rpm for 20 min. Then, an aliquot of 0.5 mL was removed from the transparent layer at the bottom of the mixture. This aliquot was reduced with 0.5 mL of 10% w/w hydroxylamine hydrochloride in 0.25 mol/L HCl for 15 min at room temperature; then, 0.5 mL of 9 mmol/L ferrozine was added. After 10 min of reaction, the absorbance at 562 nm was measured using a Biomate 3S UV–Vis spectrophotometer (Thermo Fisher Scientific, USA). The percent of EE was calculated according to Equation (3):

(3) $$EE\;\left(\%\right)=\left(\left(A_{max}-A_{x}\right)/\left(A_{max}-A_{min}\right)\right)\times 100$$

where EE (%) is the percentage encapsulation efficiency; A_max_ is the absorbance found in emulsions with the maximum iron in the W_2_ phase; A_x_ is the measured absorbance; A_min_ is the measured absorbance when there is no iron in the emulsion.

### 2.5 Particle size and zeta potential

The hydrodynamic diameter in nanometers (d.nm) of the emulsion droplets and zeta potential of the emulsions were obtained using a Zetasizer Nano ZS90 (Malvern Instruments, UK) equipped with a He-NE laser at 25 °C. The samples (A7, A8, A16, and A17) were analyzed at time zero and 07 days of storage following the methodology from Simiqueli et al. (2019).

### 2.6 Microscopy

An aliquot of the double emulsion sample A17 was placed between the slides and observed under an optical microscope (K220, Kasvi, Brazil), which was coupled with a Moticam camera (5 MP, Kasvi, Brazil) at 40x magnification using immersion oil.

### 2.7 Interfacial tension

The interfacial tension between the continuous aqueous phase of samples A7, A8, A16, and A17 and the oil phase dispersed in the solution was determined by adapting the methodology reported by Fernandes and Garcia-Rojas (2021). The crescent drop technique was used in the Easytrack tensiometer (Teclis Scientific, France) with the Easytrack® software (Teclis Instruments, France), and the analysis was performed for 3000 s. The aqueous phase was diluted 1:7 in ultrapure water, and measurements were conducted at a controlled temperature of 25 ± 0.1 °C in a CD-BC4 thermostatic bath (Julabo, Germany). The model (Eq. (4)) proposed by Shi et al. (2004) was fitted to the data obtained from the interfacial tension between systems A7, A8, A16, and A17 and the dispersed oil phase.

(4) $$\sigma \left(t\right)=\sigma_{f}+\text{exp}\left(-t/\tau_{1}\right)\left(\sigma_{1}-\sigma_{f}\right)+\text{exp}\left(-t/\tau_{2}\right)\left(\sigma_{2}-\sigma_{f}\right)$$

where *σ*_*f*_ is asymptotic interfacial tension (mN.m) for t → ∞, *t* is time in seconds, *σ*_*1*_ and *σ*_*2*_ are kinetic parameters of interfacial tension, τ_1_ is the migration time (s) of the emulsifier at the interface, and τ_2_ is the time(s) of reorganization and eventual changes in macromolecules in the interface.

### 2.8 In vitro gastrointestinal simulation of double emulsions

#### 2.8.1 Simulation of in vitro digestion in adults

Simulated salivary fluid (SSF), gastric fluid (SGF), and intestinal fluid (SIF) were prepared according to the INFOGEST 2.0 methodology (Brodkorb et al., 2019). All simulations were performed using a shaker TE-424 (Tecnal, Brazil) at 37 °C and 90 rpm.

The simulation of adult digestion started in the oral phase, where 0.7 g of emulsion sample A17 was mixed with 0.56 mL of SSF, 3.5 μL of 0.3 mol/L CaCl_2_, 70 μL of amylase solution (75 U/mL), and 67 μL of water and incubated for 2 min.

Subsequently, for gastric digestion, 1.12 mL of SGF, 0.14 mL of pepsin (2000 U/mL), and 0.7 μL of 0.3 mol/L CaCl_2_ were added to the oral digested mixtures. Then, the pH of the mixture was adjusted to 3 with HCl, and water was added to complete 2.8 mL. The gastric simulation was performed for 2 h.

Finally, intestinal digestion was performed for 2 h, and the simulated gastric digestion was mixed with 1.19 mL of SIF, 5.6 μL of 0.3 mol/L CaCl_2_ solutions, 0.7 mL of 100 U/mL pancreatin solutions, and 0.35 mL of a solution of 10 mmol/L porcine bile. The pH was adjusted to 7.0, and water was added to obtain 5.6 mL of the total solution.

#### 2.8.2 Simulation of in vitro digestion of infants

The digestion simulation was performed using the protocol by Ménard et al. (2018), and simulated gastric fluid (SGF) and intestinal fluid (SIF) systems were formed using the INFOGEST 2.0 methodology (Brodkorb et al., 2019).

The infant digestion simulation started in the gastric phase. For that, 1.76 g of emulsion sample A17 was incubated with 0.76 mL of SGF and 0.14 mL of a pepsin solution (268 U/mL). The pH of the mixture was adjusted to 5.3 with 6 mol/L HCl, and water was added to form 2.8 mL of solution. Then, 0.66 mL of SIF, 5 μL of CaCl_2_ (0.3 mol/L), 0.510 mL of pancreatin solution (90 U/mL), and 0.09 mL of porcine bile (3.1 mmol/L) were added to the intestinal sham, the pH was adjusted to 6.6, and water was added to form 4.52 mL of the total solution. All infant simulated digestion was performed in a shaker at 90 rpm and 37 °C for 2 h.

#### 2.8.3 In vitro release of iron

The in vitro release of iron after in vitro gastrointestinal simulation for infants and adults was determined by calculating its concentration at specific times (2, 10, 30, 60, 90, 120, 150, 180, 210, and 240 min) according to the adapted ferrozine chelation methodology of Choi et al. (2009) and Prichapan et al. (2021). For that, aliquots of 0.2 mL of digested mixtures were centrifuged in a Digicen 21R centrifuge (OrtoAlresa, Spain) at 8000 *g* for 5 min. Afterwards, 0.1 mL was removed from the transparent layer and added to 0.4 μL of ultrapure water and 0.5 mL of 10% w/w hydroxylamine hydrochloride. This mixed solution was allowed to react for 15 min at room temperature. Then, 0.5 mL of ferrozine (9 mmol/L) was added to the solution; after 10 min, the absorbance of the solution was measured in a Biomate 3S spectrophotometer. The release was defined by Equation (5):

(5) $$Release\left(\%\right)=\left(1-\left(\left(A_{max}-A_{x}\right)/\left(A_{max}-A_{min}\right)\right)\right)\times 100$$

where A_max_ is the absorbance in emulsions that were formed with iron at maximum concentration only in the A_2_ phase, A_x_ is the measured absorbance, and A_min_ is the measured absorbance when there is no iron in the emulsion.

### 2.9 In vitro bioaccessibility of iron

The in vitro bioaccessibility of A17 iron content was determined according to the methodology of Ilyasoglu Buyukkestelli and El (2019). After complete digestion, all samples were centrifuged with a Digicen 21R centrifuge at 8000 *g* for 5 min to separate the aqueous phase and oil phase of the digested solution. Then, an aliquot of 0.5 mL of the aqueous phase was taken for absorbance reading according to the method described for the in vitro release of iron (Section 2.8.3). The bioaccessibility of iron (B*) was calculated using Equation (6):

(6) $$\text{B*}=\left(C_{\mathrm{Micelle}}/C_{\mathrm{Digesta}}\right)\text{x}100$$

where *C*_*digesta*_ is the iron concentration in the digesta, and *C*_*micelle*_ is the iron concentration in the micellar fraction after every gastrointestinal simulation.

### 2.10 Statistical analysis

One-way analysis of variance was used to statistically analyze the results. Tukey's test was used to verify differences between samples, which were considered significant when the p value was less than 0.05. The experiments were performed in triplicate; therefore, the results are expressed as the mean ± standard deviation.

## 3 Results and discussion

### 3.1 Stability kinetics of the emulsion

**Fig. 1:**
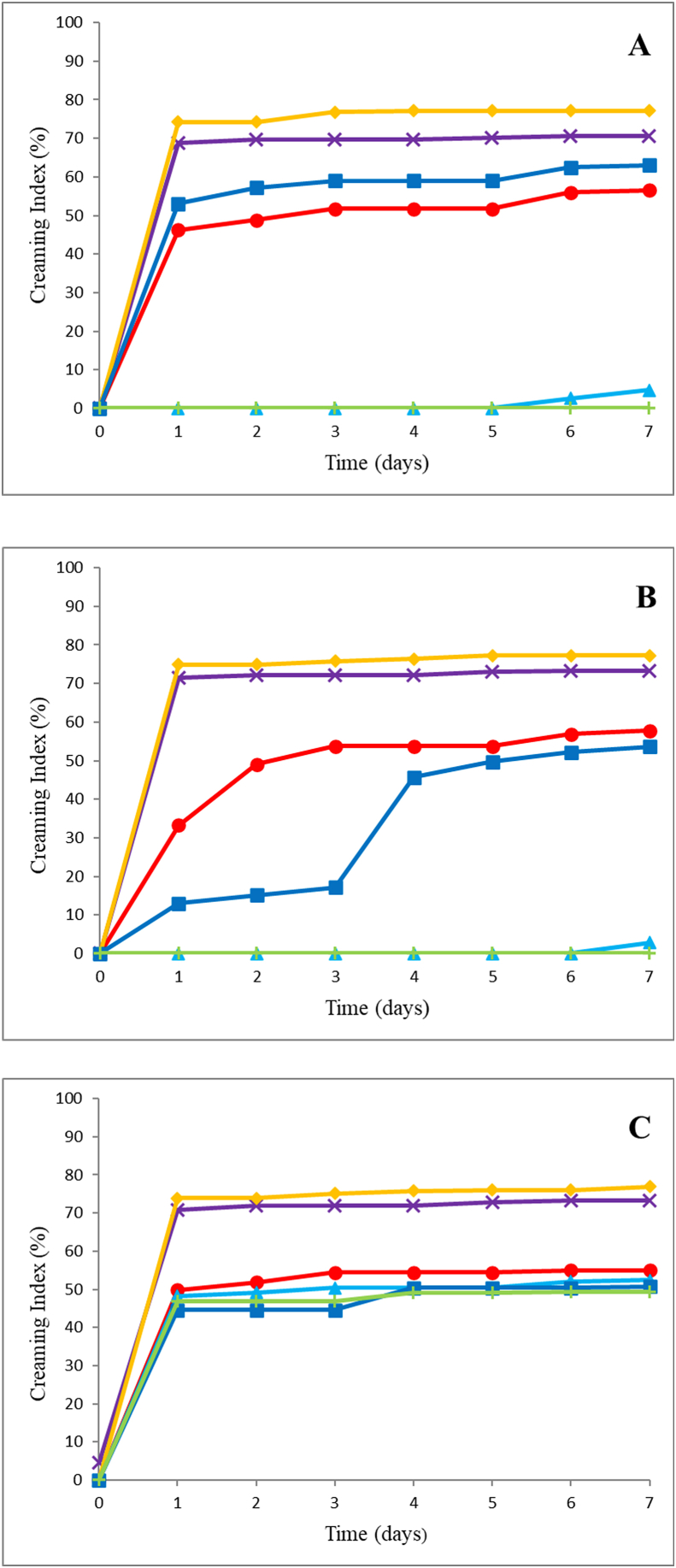
Creaming index of the different samples of double emulsions (W_1_/O/W_2_) where (A), (B) and (C) are the samples with 8%, 12% and 16% WPI, respectively. (**x**) 0% tara gum and 0% sucrose, (●) 0.4% tara gum and 0% sucrose, (▲) 0.8% tara gum and 0% sucrose, (♦) 0% tara gum and 2% sucrose, (■) 0.4% tara gum and 2% sucrose, (**+**) 0.8% tara gum and 2% sucrose.

Fig. 1 shows the creaming index of all double emulsions during 7 days of storage at 25 °C. Fig. 1 shows that the emulsions with the highest concentration of tara gum (A7, A8, A16, and A17) had the lowest creaming index during the evaluated seven days. There was no phase separation in samples A7, A8, A16 and A17 for five, six, seven and seven days, respectively. This behavior may be related to the action of tara gum as a thickening agent, since all of these samples presented a higher concentration of this gum. Tara gum has a high viscosity, which reduces the effects of gravitational instability, such as creaming (Oppermann et al., 2018; Saffarionpour and Diosady, 2021).

Emulsions A8 and A17 had a higher WPI concentration, so they formed more stable emulsions due to the interfacial action of the protein. Samples A16 and A17 did not show phase separation throughout the experiment, which may be related to the presence of sucrose, which is an OAS, improves the osmotic balance of the emulsion and prevents separation mechanisms such as Ostwald maturation (Kanouni et al., 2002; Dickinson, 2011; Klojdová et al., 2019).

Emulsions containing only WPI and sucrose (A1, A2, A3, A10, A11, and A12) had the highest creaming index, which indicates rapid phase separation. All samples with tara gum had lower creaming index values than those without tara gum, but all presented phase separation on the first day. Ilyasoglu Buyukkestelli and El (2019) found no changes in creaming index using sodium caseinate for three days in the same proportion of simple emulsion and external aqueous phase of the study. Simiqueli et al. (2019) did not obtain a creaming index greater than zero throughout the experiment for 31 days with emulsions refrigerated at 4 °C. Emulsions containing high concentrations of WPI and tara gum (A9 and A18) showed high creaming index rates, which can be explained by the hydrophilic characteristic of galactomannans, which at high tara gum concentrations (0.8%) may have contributed to the dehydration of proteins, favored their self-aggregation and consequently increased the creaming index. This effect was also reported by Dickinson and Golding (1997), who found that the creaming stability in O/W emulsions decayed with increasing concentration of sodium caseinate used as the emulsifier.

The study of kinetic stability shows that the tara gum concentration is an important factor for the stability of double emulsions. Since samples A7, A8, A16, and A17 did not present a creaming index with significant differences (p < 0.05), they were used in the following studies.

### 3.2 Encapsulation efficiency of emulsion

Table 2 shows the encapsulation efficiency (EE) on the first day immediately after the formation of the emulsion and after 7 days of storage of the double emulsions with different concentrations of WPI and sucrose. Samples A7, A8, A16, and A17 were used to obtain the EE because they presented lower index creation after 7 days of storage. From Table 2, the emulsions with a higher concentration of WPI had a higher EE. The same behavior was presented by emulsions with higher sucrose concentrations, which also presented higher EE values than those without sucrose.

**Table 2:** Encapsulation efficiency of several emulsions immediately after formed and on the seventh day of storage.

Sample	Day 0 (EE%)	Day 7 (EE%)
A7	91.9 ± 1.21 ^a^	66.1 ± 0.71 ^a^
A8	93.9 ± 0.56 ^ab^	67.1 ± 0.61 ^a^
A16	94.4 ± 0.90 ^bc^	66.0 ± 0.99 ^a^
A17	96.9 ± 1.00 ^c^	70.7 ± 0.69 ^b^

Values presented as mean ± standard deviation (n = 3), values of columns followed by the same letters do not present statistically significant difference in the Tukey test for the significance level of 5%.

After the 7th day, all emulsions significantly lost EE, which implies a transport of iron from the internal phase of the double emulsion to the external aqueous phase. The A17 emulsion showed a higher EE in the seven days of storage than the other systems. The reason may be the higher WPI concentration and the presence of sucrose in the system, which decreases the interfacial tension, improves the osmotic balance of the sample, increases the stability and hinders the transport of iron between the phases of the emulsion. These results are consistent with other studies of iron encapsulation reported in the literature. For example, Duque-Estrada et al. (2019) formulated double emulsions to encapsulate iron and observed EE variation between 88% and 96% after emulsion formation at different concentrations of PGPR, and after 7 days, their largest samples showed approximately 50% EE. In another study, Chang et al. (2016) showed an EE of 93.63% for a double emulsion formed with medium-chain triglyceride (MCT) and WPI after emulsion formation. Additionally, Hosseini et al. (2019) obtained high EE ranging from 95.33% to 54.47% for double emulsions formed with PGPR and Tween 80 as emulsifiers.

**Fig. 2:**
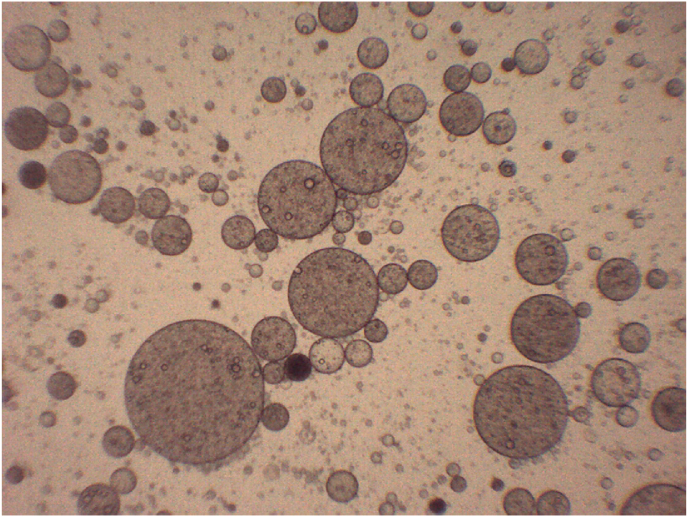
Optical microscopy image of A17 system, double emulsion W/O/W composed of 12% WPI, 0.8% tara gum and 2% sucrose in the external aqueous phase.

Fig. 2 shows the structure of the formed double emulsion (A17). We can see several drops of oil that are dispersed in the aqueous phase (W_2_). These drops are bordered by a prominent black circle. Furthermore, within these oil drops, we can have small circles with a clear appearance, which can be assumed to be the internal aqueous phase (W_1_) where the iron was encapsulated. Similar images were observed in the works by Ilyasoglu Buyukkestelli and El (2019) and Simiqueli et al. (2019).

### 3.3 Particle size and zeta potential

The particle size distribution is shown from the mean size in Table 3. The values were obtained after the emulsion formation and after 7 days of storage at 25 °C. In this table, samples with higher WPI concentrations had significantly (*p* < 0.05) lower sizes (757.1 ± 45.7 d nm for A17 and 782.8 ± 15.5 d nm for A8) than those with lower WPI concentrations (842.9 ± 2.1 d nm for A16 and 876.7 ± 116 d nm for A7). This effect was also observed by Amine et al. (2014) in pea, potato, and casein proteins. This effect may be related to the higher protein concentration, which can bind to a greater surface area and consequently decrease the average diameter of a particle. After 7 days, there was a significant increase (*p* < 0.05) in mean diameter of all samples, which ranged from 845.2 ± 7.0 nm to 922.1 ± 55.5 nm and showed the beginning of the separation processes of the phases (coalescence and flocculation).

**Table 3:** Particle size and Zeta potential of the various emulsions immediately after formed and on the seventh day of storage.

Sample	Particle size (nm)		Zeta Potential (mV)	
	Day 0	Day 7	Day 0	Day 7
A7	876.7 ± 11.6^cA^	922.1 ± 55.5 ^aB^	−44.53 ± 1.39 ^cA^	−42.73 ± 0.86 ^aB^
A8	782.8 ± 15.5 ^abA^	845.2 ± 7.0 ^aB^	−49.27 ± 0.35 ^aA^	−42.23 ± 1.15 ^aB^
A16	842.9 ± 2.1 ^bcA^	891.2 ± 39.3 ^aB^	−46.90 ± 0.75 ^bA^	−42.83 ± 0.50 ^aB^
A17	757.1 ± 45.7 ^aA^	856.8 ± 11.6 ^aB^	−49.67 ± 0.15 ^aA^	−41.30 ± 0.61 ^aB^

Values presented as mean ± standard deviation (n = 3), values followed by the same letters do not present statistically significant difference in the Tukey test for the significance level of 5%. Lowercase letters match samples and uppercase letters match time.

Table 3 shows the zeta potentials measured immediately after the formation of the systems and after 7 days of storage at 25 °C. It is possible to identify a high negative charge density in the systems, ranging from −44.53 ± 1.39 mV to −49. 67 ± 0.15 mV on the first day. High charge densities demonstrate the high stability kinetics of the emulsion due to the repulsion among the internal emulsion droplets in the double emulsion. This phenomenon occurs because whey proteins are negatively charged, and when they bind the oil surface, they form structures that repel one another (McClements, 2004). High negative charges in double emulsions were also identified by Simiqueli et al. (2019), who found zeta potentials ranging from −20.9 mV to −50.1 mV in different samples. Prichapan et al. (2021) found a zeta potential of −46 mV when they formed double emulsions using quillaja saponin. After 7 days, the zeta potential of the samples ranged from −42.73 ± 0.86 mV to 41.3 ± 0.61 mV. This significant drop (p < 0.05) characterizes a reduction in the stability of the samples, which was also observed in the particle size distribution.

### 3.4 Interfacial tension

**Fig. 3:**
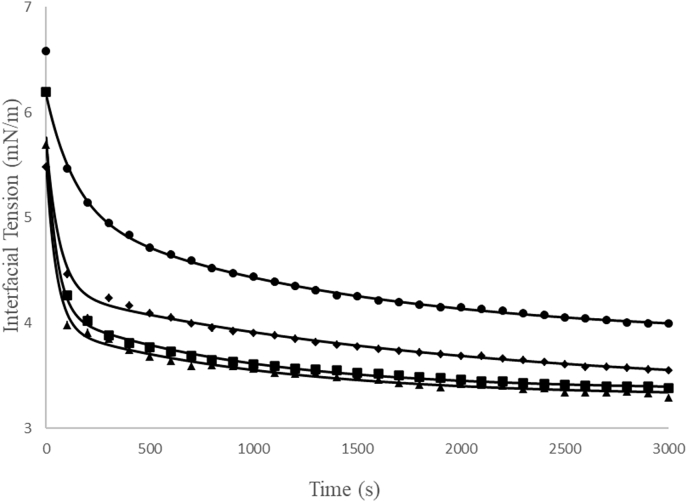
Dynamic interfacial tension of the emulsion samples and their respective models. (●) sample A7; (■) sample A8; (♦) sample A16; (▲) sample A17 and (– –) model by Equation (4).

Fig. 3 shows the variation in the dynamic interfacial tension between the simple emulsion and the aqueous phases of systems A7, A8, A16, and A17 as a function of time. Table 4 presents the quantitative values of different parameters of adjustment of Equation (4) in different samples. The studied model presented a good fit to the experimental data of the evaluated samples with adjusted R^2^ values close to 1.0, a low mean absolute deviation (AAD), a higher value of 0.53%, and a standard deviation (SD) not greater than 0.04 mN/m.

**Table 4:** Parameters of dynamic interfacial tension between the external aqueous phase and the simple emulsion estimated from Equation 4.

	σ_f_ (mN/m)	σ_1_ (mN/m)	σ_2_ (mN/m)	τ_1_ (s)	τ_2_ (s)	AAD∗ (%)	SD∗∗ (mN/m)
A7	3.88 ± 0.01	4.98 ± 0.03	5.05 ± 0.02	138.37 ± 6.29	1324.24 ± 33.46	0.30	0.03
A8	3.37 ± 0.01	5.03 ± 0.03	4.09 ± 0.01	48.77 ± 1.53	927.76 ± 28.14	0.46	0.04
A16	3.36 ± 0.01	4.78 ± 0.05	4.29 ± 0.01	62.86 ± 2.26	1915.20 ± 59.80	0.41	0.04
A17	3.31 ± 0.01	4.87 ± 0.04	3.95 ± 0.01	48.91 ± 1.47	1050.11 ± 37.65	0.53	0.03

σ_exp_ is experimental interfacial tension (mN.m), σ_cal_ is the tension calculated by Equation (4) (mN.m), m is the number of experimental points and p *is* the number of parameters adjusted.

∗ $AAD\left(\%\right)=\left(\frac{100}{m}\right)\left[\overset{m}{\underset{i=1}{\sum }}\frac{\sigma_{exp,i}-\sigma_{cal,i}}{\sigma_{exp,i}}\right]\;;$

∗∗ $SD=\sqrt{\frac{\sum_{i=1}^{m}{\left(\sigma_{exp,i}-\sigma_{cal,i}\right)}^{2}}{m-p}}$

The A17 system obtained a lower interfacial tension (σf) of 3.31 ± 0.01 mN/m because of its higher protein concentration, which generated a higher action at the oil-water interface and consequently a lower interfacial tension. The reason is that the protein-water and protein-oil interactions are thermodynamically more favorable, so they increase the system stability and hinder separation mechanisms such as Ostwald maturation (McClements, 2004). These results are consistent with the results of the kinetic stability of the emulsions (Section 3.1.), since the A17 emulsion did not present phase separation during 7 days of evaluation, while sample 7, which has a tension of 3.88 ± 0.01 mN/m, showed phase separation on its 5th day. Amine et al. (2014) also observed a decrease in interfacial tension with increasing concentrations of pea, potato, and sodium caseinate proteins.

Samples A8 and A17 have shorter emulsifier migration time to the interface (τ1) (48.77 ± 1.53 s and 48.91 ± 1.47 s, respectively) and shorter macromolecule reorganization time (τ2) (927.76 ± 28.14 s and 1050.11 ± 37.65 s, respectively), which can be explained by the higher protein concentrations of these systems. Mahfoudhi et al. (2014) also observed that with increasing concentrations of the emulsifier (almond gum), parameters τ_1_ and τ_2_ decreased. In another study, Donsì et al. (2012) observed that increasing the emulsifier Tween 80 at a certain concentration decreased the values of τ_1_ and τ_2_.

### 3.5 In vitro digestion of the double emulsion containing iron

**Fig. 4:**
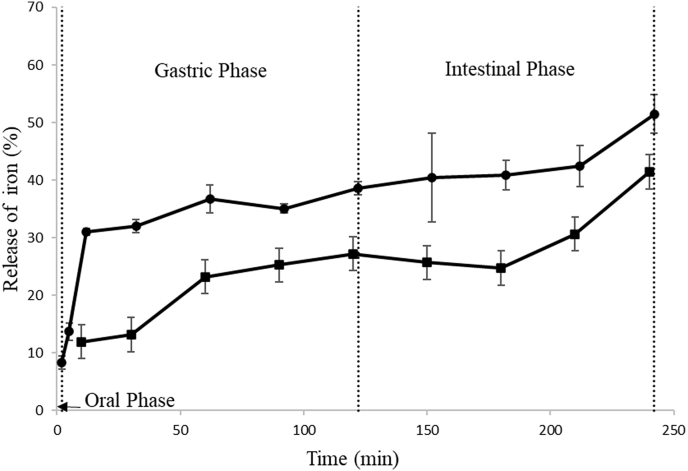
Iron release in the gastrointestinal simulations of sample A17 as a function of time. (●) adult simulation and (■) infant simulation.

Fig. 4 shows the iron release from the double emulsion (A17) during the simulation of the in vitro digestion of both adults and infants at different times. For adult digestion, 8.36% of the encapsulated iron was released in the oral phase, which may be attributed to the dilution (1:1 ratio) of the emulsion with salivary fluid.

In the gastric phase, the release increased and reached 38.56% at the end. The high release of iron in the gastric phase was attributed to the action of pepsin at a strong acid phase, which converted proteins in peptides and amino acids, affected the molecules of WPI that were linked to the interface, decreased the repulsion among the droplets of the emulsion, caused separation processes and released iron.

Finally, the intestinal phase ended with a total release of 51.47%; this release increased in the intestinal phase due to the presence of lipase, which breaks down fat molecules of the oil phase (soybean oil) into free fatty acids and monoacylglycerols to release iron (McClements, 2014). Tara gum is an indigestible polysaccharide; thus, it is not hydrolyzed by enzymes in the human body (Wüstenberg, 2014). Hosseini et al. (2019) observed a release of 55.62% of iron in emulsions after the intestinal phase.

Digestion in infants only occurs in the gastric and intestinal phases because the oral phase is not necessary, since it is a simulation of infants who cannot yet chew and directly pass all food into the esophagus. Therefore, 27.22% and 41.45% release of the encapsulated iron was observed at the end of the gastric and intestinal phases, respectively. The adult simulation showed a significantly (p < 0.05) higher iron release rate in the gastric and intestinal phases because digestion in infants is a much milder process than digestion in adults. Furthermore, pepsin and lipase activities are lower in infants, and the pH of the simulated gastric phase of the infants is also very different from that of the adult phase (5.3 and 3.0, respectively), which reduces the effectiveness of pepsin, which works better at more acidic pH values. Liu et al. (2018) also observed greater release in sham systems for adults than sham systems for infants using lactoferrin-loaded liposomes. These authors observed that after complete simulation, infant samples obtained 20.1% intact liposomes, while adult samples obtained less than 20% intact liposomes.

### 3.6 Bioaccessibility

After the in vitro stimulation of gastrointestinal digestion, the bioaccessibility of iron was 49.54% for adults and 39.71% for infants. Bioaccessibility indicates the amount of iron that is soluble and ready to absorb by the digestive system (Dima et al., 2020; McClements and Peng, 2020). Major factors that influence this solubility are the chemical form of iron and the presence of inhibitors (Da Silva et al., 2013; Bryszewska, 2019). Soluble iron is absorbed by the human body in the duodenum of the small intestine at neutral pH in both haem and non-haem forms, where the haem form is better absorbed. Ferrous iron is the only form of non-haem iron absorbed by the intestine and can be transformed to haem molecules, stored, or transported to sites where iron is required (Hunt, 2005; Shubham et al., 2020). Infants and adults had significantly different results (p < 0.05), and the bioaccessibility of infants was approximately 20% lower than that of adults. This difference can be explained by the pH values and enzymatic activity that affected digestion, as discussed in Section 3.5. Marques et al. (2021) also observed differences for infants and adults. The authors observed that the bioaccessibility of docosahexaenoic acid varied by 12–25% between treatments based on simulations for adults and simulations for infants, following the same methodologies of Ménard et al. (2018) and INFOGET 2.0 (Brodkorb et al., 2019). In the case of bioaccessibility in adults, other studies were reported in the literature, such as that by Ilyasoglu Buyukkestelli and El (2019), who obtained the bioaccessibility of iron ranging from 41.17% to 52.97% depending on the ratio between the simple emulsion in the double emulsion studied. Hosseini et al. (2019) reported that the bioaccessibility of iron in double emulsions was 37.28–42.94% depending on the heat treatment used.

## 4 Conclusions

In this study, it was possible to obtain stable double emulsions using WPI and PGPR as emulsifying agents and tara gum as a thickening agent. The presence of tara gum ensured an increase in general stability of the emulsion, which prevented gravitational separation mechanisms. Sample A17 (12% WPI, 0.8% tara gum, and 2% sucrose) formed stable double emulsions for 7 days with high iron encapsulation efficiency. The in vitro simulations of gastrointestinal digestion show that the ideal system obtained a high bioaccessibility of iron for adults and infants. This study identified that double emulsions could present themselves as stable solutions and with a high bioaccessibility for iron, even in mild digestive systems such as that of infants. These studied emulsions are presented as a potential alternative for use in iron-fortified food liquid formulations such as milk and other dairy products for both infants and adults.

## CRediT authorship contribution statement

**Bruno Sérgio Toledo Barbosa:** Conceptualization, Methodology, Validation, Investigation, Writing – original draft, Writing – review & editing. **Edwin Elard Garcia-Rojas:** Conceptualization, Methodology, Resources, Writing – review & editing, Supervision, Project administration, Funding acquisition.

## Declaration of competing interest

The authors declare that they have no known competing financial interests or personal relationships that could have appeared to influence the work reported in this paper.
